# Supplementary material for: O-Linked Sialoglycans Modulate the Proteolysis of SARS-CoV-2 Spike and Likely Contribute to the Mutational Trajectory in Variants of Concern
Source: ACS Cent Sci. 2023 Feb 16;9(3):393–404. doi: 10.1021/acscentsci.2c01349 (PMC10037455; doi:10.1021/acscentsci.2c01349)
Supplement: Supplementary file 4 — oc2c01349_si_004.pdf [file oc2c01349_si_004.pdf]

oc-2022-01349s.R1

Name: Peer Review Information for "O-Linked Sialoglycans Modulate the Proteolysis of SARS-CoV-2 Spike and Likely Contribute to the Mutational Trajectory in Variants of Concern"

First Round of Reviewer Comments

Reviewer: 1

Comments to the Author

In this work, Gonzalez-Rodriguez, et al, investigated the influences of the O-linked sialoglycans on the stability of the SARS-CoV-2 spike protein. The N-terminal region to the polybasic furin-cleavage site R682RAR685 was targeted. Specifically, the O-GalNAc glycans on Thr678 were found to play a crucial role in proteolytic cleavage of the polybasic cleavage site. This work provides significant insights into our understanding of the unique presence of the polybasic cleavage sites in SARS-CoV-2. I do find that the literature review is incomplete. I'd like to recommend the publication after the authors provide a more complete review of the related studies.

The one below is possibly the very first paper on the role of the polybasic cleavage site of SARS-CoV-2

1) Walls, A. C.; et al.,. Structure, Function, and Antigenicity of the SARS CoV-2 Spike Glycoprotein. Cell 2020, 181, 281−292.

.

The following is one computer simulation paper on the polybasic cleavage site.

2) Qiao & Olvera de la Cruz, Enhanced Binding of SARS-CoV-2 Spike Protein to Receptor by Distal Polybasic Cleavage Sites, ACS Nano 2020, 14, 8, 10616–10623

Reviewer: 2

Comments to the Author

In this manuscript, the authors report that a disruption of O-GalNAc glycosylation on spike protein potentially acts as a major factor for evolution of variants of concern. A unique combination of bump-and-hole engineering tool and MS-based approach has been used to identify individual glycosyltransferase GalNAc-T1 derived sialoglycans on spike protein that showed substantial reduction of furin activity as well as impacted spike cleavage by TMPRSS2. The manuscript is well developed and can be accepted for publication after minor revisions:

1. It would be good to have a few lines in the introduction on the fragmentation types used in MS-based methods and advantages of using ETD over just HCD.

2. Figure 1A right shows the FCS regions for SARS-CoV-1 as well, is there any comparisons made between SARS-CoV-1 and 2 using the chemical tool?

3. The observations made on PBMCs form an important finding which should be brought to the main text. The evaluation of n=48 vaccinated individuals – were there any controls here?

4. Figure 3B and text – what do you mean by starting material? A short table or description on the different panel will be helpful for readers.

5. Again, a short table describing the different FRET is easier to follow – maybe as a panel in the figure itself. Please also explain the rationale behind the development of FRET1 to 9?

6. Please clarify what the different symbols are in the bar graphs of fig 5.

7. Fig 6 uses recombinant spikes – which cell cultures were used? Could the alterations occur due to differences in expression system?

8. Why is there another “discussion” header after “result and discussion”?

Author's Response to Peer Review Comments:

Dear Prof. Editor,

Thank you for your encouraging decision letter informing us of the positive reviews on our article "O-Linked Sialoglycans Modulate the Proteolysis of SARS-CoV-2 Spike and Likely Contribute to the Mutational Trajectory in Variants of Concern".

We have addressed the reviewers’ comments in the documents I am uploading. We have also prepared a proposed Journal Cover artwork.

Thank you for your time and for considering our article for ACS Central Science.

Sincerely,

Dr Benjamin Schumann

Dr Benjamin Schumann
Imperial College London
and The Francis Crick Institute
1 Midland Road, London, NW1 1AT
b.schumann@imperial.ac.uk

Response to reviewers:

<u>*Reviewer 1*</u>

Comments:

In this work, Gonzalez-Rodriguez, et al, investigated the influences of the O-linked sialoglycans on the stability of the SARS-CoV-2 spike protein. The N-terminal region to the polybasic furin-cleavage site R682RAR685 was targeted. Specifically, the O-GalNAc glycans on Thr678 were found to play a crucial role in proteolytic cleavage of the polybasic cleavage site. This work provides significant insights into our understanding of the unique presence of the polybasic cleavage sites in SARS-CoV-2. I do find that the literature review is incomplete. I'd like to recommend the publication after the authors provide a more complete review of the related studies.

The one below is possibly the very first paper on the role of the polybasic cleavage site of SARS-CoV-2:

1) Walls, A. C.; et al., Structure, Function, and Antigenicity of the SARS CoV-2 Spike Glycoprotein. Cell 2020, 181, 281−292.

The following is one computer simulation paper on the polybasic cleavage site:

2) Qiao & Olvera de la Cruz, Enhanced Binding of SARS-CoV-2 Spike Protein to Receptor by Distal Polybasic Cleavage Sites, ACS Nano 2020, 14, 8, 10616–10623.

***A**: We thank the reviewer for their comments and agree that the suggested references are an essential addition to the referenced literature. We have thus included the references to the revised manuscript.*

<u>*Reviewer 2*</u>

Comments:

In this manuscript, the authors report that a disruption of O-GalNAc glycosylation on spike protein potentially acts as a major factor for evolution of variants of concern. A unique combination of bump-and-hole engineering tool and MS-based approach has been used to identify individual glycosyltransferase GalNAc-T1 derived sialoglycans on spike protein that showed substantial reduction of furin activity as well as impacted spike cleavage by TMPRSS2. The manuscript is well developed and can be accepted for publication after minor revisions:

1. It would be good to have a few lines in the introduction on the fragmentation types used in MS-based methods and advantages of using ETD over just HCD.

***A**: A paragraph describing the advantages of electron-based dissociation methods (like ETD) for glycan analysis was incorporated to the revised manuscript:*

"While collisional fragmentation (i.e., higher energy collision dissociation, HCD) allows for the determination of monosaccharide compositions and naked peptide backbone sequences, this technique does not allow for the localization of O-glycans to their glycosites. The energy associated with collisional dissociation methods breaks the most labile bonds, which in the case of glycopeptides, is the glycosidic linkages between monosaccharides and the connection of the glycan to the peptide itself. To resolve site information in O-glycopeptides, electron-based dissociation methods must be employed; commonly this involves electron transfer dissociation (ETD)"

2. Figure 1A right shows the FCS regions for SARS-CoV-1 as well, is there any comparisons made between SARS-CoV-1 and 2 using the chemical tool?

***A**: We have no experimental data that directly compares the FCS proximal region of SARS-CoV-2 and the analogous region in SARS-CoV. We performed an in-silico analysis using the NetOGlyc-4.0—which produces neural network predictions of mucin type GalNAc O-glycosylation sites in mammalian proteins—for SARS-CoV and found low prediction scores for Thr662 (score = 0.0583402), Ser668 (score = 0.415689), and Thr669 (score = 0.323025). Since the sequence of that peptide region is so different between SARS-CoV and SARS-CoV2, we would refrain from any conclusions of the impact O-glycosylation between the two.*

3. The observations made on PBMCs form an important finding which should be brought to the main text. The evaluation of n=48 vaccinated individuals – were there any controls here?

***A**: The PBMC findings are discussed in the main text (last paragraph before "GalNAc-T selective MS-glycoproteomics analysis allows O-glycosite and glycan composition investigation in vitro and in engineered cells" section).*

The n=48 vaccinated individuals were all from the Legacy study and therefore essentially 'healthy controls', no convalescent samples from patients with SARS-CoV-2 in hospital were tested. Since the goal was to evaluate if the glycopeptides elicited an immune response in individuals with some level of immunity towards the virus, there were no unvaccinated controls.

4. Figure 3B and text – what do you mean by starting material? A short table or description on the different panel will be helpful for readers.

***A**: In line with the reviewer's comments and to clarify the outcome of the glycosylation reactions from figure 3B, we have changed the bar description from "Starting material" to "Non-glycosylated". Additionally, we have expanded the figure legend for figure 3A, which intends to show what the 10 peptides that were subjected to the chemoenzymatic glycosylation by WT-T1 were, as well as the general components of this reaction.*

5. Again, a short table describing the different FRET is easier to follow – maybe as a panel in the figure itself. Please also explain the rationale behind the development of FRET1 to 9?

***A**: We appreciate that including details on the logic behind the target glycopeptides upon their introduction to the text substantially aids the reader following this section while enriching the discussion of the corresponding results. We have therefore included details on the rationale behind the design of all glycopeptides (FRET-1 through FRET-9) to the revised discussion.*

6. Please clarify what the different symbols are in the bar graphs of fig 5.

***A**: The missing symbol information for figure 5 has been added to the figure legend in the revised manuscript.*

7. Fig 6 uses recombinant spikes – which cell cultures were used? Could the alterations occur due to differences in expression system?

***A**: We can rule out any expression variabilities since both analysed fractions come from the same protein preparation. One fraction is the uncleaved FL-S and the other the cleaved S1/S2 component of the same purified recombinant SARS-CoV-2 S preparation. FL-S and S1/S2 were separated by SDS-PAGE and separately subjected to the glycoproteomics workflow.*

We now appreciate that this is not clearly stated in the manuscript and could easily cause confusion. For clarity's sake, we have rephrased the paragraph discussing these results in the revised manuscript as follows:

"After SDS-PAGE, in-gel digestion allowed us to directly test this notion by individually cutting out the bands corresponding to the cleaved (S1/S2) and uncleaved (FL-S) fractions from the same recombinant WT-spike preparation. We subjected the FL-S and S1/S2 gel bands to MS-glycoproteomics analysis..."

8. Why is there another “discussion” header after “result and discussion”?

***A**: Changed the title of the last section from "Discussion" to "Conclusions"*

oc-2022-01349s.R2

Name: Peer Review Information for "O-Linked Sialoglycans Modulate the Proteolysis of SARS-CoV-2 Spike and Likely Contribute to the Mutational Trajectory in Variants of Concern"

Second Round of Reviewer Comments

Reviewer: 1

Comments to the Author

My comments have been addressed.

Reviewer: 2

Comments to the Author

The authors have addressed my concerns appropriately.

Author's Response to Peer Review Comments:

Dear Dr. Editor,

thank you so much for your encouraging e-mail, suggestion a re-submission after addressing the formatting needs. We have addressed these in the accompanying submission. We are also providing a suggestion for a cover image.

Best regards,

Benjamin Schumann
